# Prediabetes Is Associated with *HNF-4***α**** P2 Promoter Polymorphism rs1884613: A Case-Control Study in Han Chinese Population and an Updated Meta-Analysis

**DOI:** 10.1155/2014/231736

**Published:** 2014-10-15

**Authors:** Changyi Wang, Sihan Chen, Tao Zhang, Zhongwei Chen, Shengyuan Liu, Xiaolin Peng, Jianping Ma, Xiaohong Zhong, Yanqiong Yan, Linlin Tang, Yifeng Mai, Liyuan Han, Shiwei Duan

**Affiliations:** ^1^Shenzhen Nanshan Center for Chronic Disease Control, Shenzhen 518054, China; ^2^Shenzhen Shekou People's Hospital, Shenzhen 518067, China; ^3^The Affiliated Hospital, School of Medicine, Ningbo University, Ningbo, Zhejiang 315000, China; ^4^School of Medicine, Zhejiang Provincial Key Laboratory of Pathophysiology, Diabetes Center, Ningbo University, Ningbo 315211, China

## Abstract

*Background*. Controversy remains for the association between hepatocyte nuclear factor *4α* (*HNF-4α*) P2 promoter polymorphism rs1884613 and type 2 diabetes (T2D). There was no association test of this polymorphism with prediabetes and T2D in the Chinese population. Moreover, an updated meta-analysis in various ethnic groups is needed to establish the contribution of rs1884613 to T2D risk. *Methods*. Using the Sequenom MassARRAY platform approach, we genotyped rs1884613 of *HNF-4α* in the P2 promoter region among 490 T2D patients, 471 individuals with prediabetes, and 575 healthy controls. All the individuals were recruited from 16 community health service centers in Nanshan district in Shenzhen province. Using STATA 11.0 software, meta-analysis was performed to summarize the overall contribution of rs1884613 to T2D risk. *Results*. Polymorphism rs1884613 was associated with genetic susceptibility to prediabetes in the whole samples (OR = 1.40, 95% CI = 1.16–1.68, P = 0.0001) and the female subgrouped samples (OR = 1.48, 95% CI = 1.14–1.92, P = 0.003) after adjusting for age and body mass index (BMI). In contrast, there was no association of rs1884613 with T2D in the whole samples and male in our case-control study and meta-analysis. *Conclusions*. Our results suggest that rs1884613 contributes to susceptibility to prediabetes, whereas this polymorphism may not play an important role in the development of T2D.

## 1. Introduction

The prevalence rate of type 2 diabetes (T2D) is increasing rapidly, with 8.4% of the global prevalence rate of the adults according to the International Diabetes Federation [1]. T2D is a complex metabolic disorder with strong familial aggregation and genetic predisposition [2]. Although genetic studies have made substantial progress in exploring the roles of genes in the development of T2D, the current findings only explain a small proportion of heritability [3, 4]. Meanwhile, controversies remain for the validation of candidate genes in diverse populations with different genetic backgrounds.

As a pivotal transcription factor in the progress and functions of the pancreatic *β*-cells, hepatocyte nuclear factor* 4α* (*HNF-4α*) is differentially expressed in many tissues including the liver and pancreas [5].* HNF-4α* spans 29 kb on chromosome 20q13.1-13.2 [6], which is a known T2D susceptibility locus.* HNF-4α* was shown to play a significant role in the development of maturity-onset diabetes of the young-1, which is characterized by impaired insulin secretion [7].

Polymorphism rs1884613 is located in the* HNF-4α* P2 promoter, which is about 45.6 kb upstream of the transcription start site and regulates the primary splicing form of* HNF-4α* in the pancreatic *β*-cell [8, 9]. Strong association was found between the haplotype of P2 promoter variants and T2D risk; however no significant associations for the haplotypes of other variants outside the P2 promoter region were found, suggesting that the P2 promoter region might be the susceptibility region for T2D [10].

P2 promoter polymorphism rs1884613 of* HNF-4α* was tested with T2D susceptibility in various ethnicities but not in the Chinese Han population. Considering the diverse genetic background among different populations, for which they may have a different predisposing genetic risk, the influence of rs1884613 on T2D susceptibility in the Chinese population remained to be identified. Therefore we studied the association of rs1884613 among 490 T2D subjects, 471 prediabetes subjects, and 575 healthy controls to extend knowledge in different populations.

Winckler et al. demonstrated the significant association of rs1884613 in the P2 promoter region in >3,400 patients and controls from Sweden, Finland, and Canada; however they did not confirm the significant association in an additional sample of 4,470 patients from North America and Poland (trended in the opposite direction) [11]. The inconsistencies across studies may be due to the lack of power and inadequate sample size among individual studies; therefore it is necessary to conduct a comprehensive meta-analysis to confirm the relevant relationship. In addition to the present case-control study, we also conducted an updated meta-analysis in several ethnic groups with all published studies and our case-control data.

## 2. Research Design and Methods

The participants in the current study came from 16 community health service centers (CHSC) in Nanshan district under the supervision of Shenzhen Nanshan Center for Chronic Disease Control. We applied a two-stage sampling method and a simple random procedure according to the sequence of computer-generated random numbers. Among those 1516 subjects, 490 had T2D (242 men and 248 women, mean age: 62.76 ± 11.14 years, mean body mass index (BMI): 24.95 ± 3.46 kg/m^2^), 471 were prediabetes subjects (230 men and 241 women, mean age: 61.39 ± 11.43 years, mean BMI: 25.28 ± 3.82 kg/m^2^), and 575 were healthy controls (286 men and 289 women; mean age: 57.94 ± 10.81 years; mean BMI: 23.52 ± 3.17 kg/m^2^).

T2D and prediabetes were diagnosed according to the criteria of the American Diabetes Association guidelines in 2010 [12]. Informed written consent was obtained from all subjects before participation. The study was approved by the Ethical Committee of Shenzhen Nanshan Center for Chronic Disease Control.

## 3. Genotyping

The blood samples (5 mL) were collected after overnight fasting at morning without stasis in EDTA vacutainers. Polymorphism rs1884613 was genotyped using Sequenom's MassARRAY iPLEX system according to the manufacturers' instructions. Primers for the PCR and single base extension were designed using the Sequenom software. DNA sequences of primers were 5′-ACGTTGGATGACTCTGTCGTGGCTCCAGTA-3′ and 5′-ACGTTGGATGAATTGGCTTGTGGACATCCG-3′. Thermocycling was carried out under the following conditions: 94°C for 15 min followed by 45 cycles at 94°C for 20 s, 56°C for 30 s, and 72°C for 1 min, with a final incubation at 72°C for 3 min.

## 4. Statistical Analysis

The age and BMI of involved individuals were described as means ± SD. The differences of age and BMI among T2D subjects, prediabetes subjects, and healthy controls were tested using one-way ANOVA. Deviation of Hardy-Weinberg equilibrium (HWE) was assessed by *χ* ^2^ test. To prevent the influence from genetic assumptions on the inheritance models, additive, recessive, and dominant models were applied for the association tests. Binary logistic regression analysis was used to calculate the odds ratios (ORs) and 95% confidence intervals (CIs) after being adjusted by age and BMI. Bonferroni's adjustment was applied to the significance thresholds, and a *P* value of <0.008 was adopted as the significant threshold (Table 2). Power analysis was simulated with the Power and Sample Size Calculation software [13]. All statistical tests were performed by SPSS program version 17.0 (SPSS, Chicago, IL).

Eligible studies were identified by searching PubMed, Embase, Cochrane Library, and Science Citation Index Expanded databases for articles published in English until July 10, 2014. We applied the following search terms: “*HNF-4α*” or “polymorphism” or “variant” or “rs1884613” and in combination with “diabetes” or “T2D.” The reference lists in the involved articles were also checked for additional relevant publications. Two investigators independently extracted the following data: the first author's name, year of publication, country, ethnicity, source of controls, number of cases and controls, the available genotype, and allele frequencies. Any disagreement was resolved by discussion. ORs with 95% CIs were estimated to assess the strength of association; the significance was determined using the *Z* test (*P* < 0.05 was considered statistically significant). Subgroup analyses were performed based on ethnicity (Caucasian or Asian) and source of control groups (population- or hospital-based studies). Heterogeneity was measured using Cochran's *Q* test and *I* ^2^ test [14]. If the heterogeneity was not significant (*P* > 0.10), the fixed-effect model was used to evaluate the summary ORs and 95% CIs. Otherwise, the random-effect model was applied. The *I* ^2^ was used to estimate the heterogeneity quantitatively, with *I* ^2^ < 25%, 25–75%, and >75% represented as low, moderate, and high degrees of inconsistency, respectively. Meta-analysis was conducted with the STATA software (version 11; Stata Corporation, College Station, Texas).

## 5. Results

The characteristics of the case-control subjects were shown in Table 1. There was statistical significance in age and BMI between prediabetes versus controls, type 2 diabetes versus controls (Table 1). As shown in Table 2, genotype distribution of rs1884613 in each subgroup met HWE. The results of genotypic association analyses were shown in an additive model, dominant model, and recessive model, respectively. The *P* values presented were corrected for multiple testing (with *P* < 0.008 as significant threshold). According to power calculations, our sample size provided > 90% power (at ∂ = 0.05) to detect the relative associations for rs1884613 with prediabetes risk.

After adjusting for age and BMI, rs1884613 showed strong association with prediabetes in the whole samples (Table 2, OR = 1.40, 95% CI = 1.16–1.68, *P* = 0.0001) and in female samples (Table 2, OR = 1.48, 95% CI = 1.14–1.92, *P* = 0.003). However, no association was found for the relationship between rs1884613 and T2D in the whole and male samples under the Bonferroni's correction (Table 2).

As reported in Table 3, the minor allele frequencies of rs1884613 were different among various ethnic samples. The allele frequencies of rs1884613-G were 0.16 and 0.18 in the Scandinavia and Norwegian populations, in contrast to 0.46 and 0.43 in the Japanese and Han Chinese populations. Inconsistent results were reported for the association of rs1884613 with T2D; therefore it is necessary to conduct a meta-analysis combined with several ethnic groups together at the same time. As shown in Table 4, the current meta-analysis involved a total of 8 studies with 8569 T2D subjects and 8528 controls. Among the 8 studies, 5 were population-based studies (including our study) [11, 15–17] and 3 were hospital-based studies [10, 18, 19]. There were 5 studies from Caucasian populations [11, 15–18] and 3 studies from Asian populations (including the present study) [10, 19] (Table 4).

**Figure 1:**
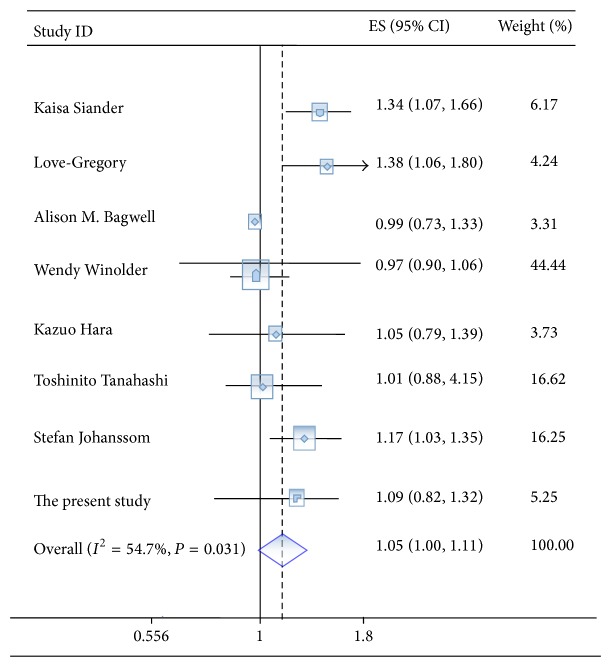
Meta-analysis of rs1884613 and type 2 diabetes risk under an additive model.

No significant association was observed in the whole meta-analysis (Table 5 and Figure 1, OR = 1.05, 95% CI = 0.99–1.11, *P* = 0.06, *P*_heterogeneity_ = 0.03, and *I* ^2^ = 54.7%). No significant associations were observed in the stratified analyses by source of controls and ethnicity (Table 5). Significant heterogeneity was found in the whole meta-analysis and the subgroup meta-analysis (White and population-based studies). The Begg's and Egger's tests were performed to assess the publication bias, and no evidence of publication bias was observed (Table 5).

## 6. Discussion

We performed a case-control study to identify the effect of rs1884613 of* HNF-4α* gene on prediabetes and T2D susceptibility in Han Chinese population. Our results suggest that rs1884613 contributes to prediabetes susceptibility in the whole and female samples. Both our case-control association and the follow-up meta-analysis are unable to support an important role of rs1884613 in the risk of T2D. Our case-control study has >90% power to detect an association with an OR of 1.40 or greater. To the best of our knowledge, this is the first report for the association of* HNF-4α* rs1884613 with prediabetes and T2D in Han Chinese population.

It should be noted that Damcott et al. also conducted a study to explore the SNPs across the promoter and coding regions of* HNF-4α* with T2D in 137 T2D, 139 prediabetes, and 342 healthy individuals; they only found that rs1884614 was significantly associated with T2D and the combined samples of T2D and prediabetes [20]. However they did not analyze the data according to gender; therefore we cannot compare the results concerning gender between our study and their study.

Consistent with our study, Winckler et al. and Bagwell et al. reported no association of* HNF-4α* polymorphisms (including rs1884613) with T2D in Scandinavians and Caucasian Americans [11, 18], although several other studies found the significant association with risk of T2D in the Finnish population [16], the Ashkenazi Jews [15], the Mexican Americans [21], and the Norwegians [17]. The opposite findings among different studies may be due to different sample size, environmental factors, and different genetic backgrounds. In the studies by Silander et al. and Love-Gregory et al., the cases were mainly from families with history of T2D, which may overestimate the contribution of rs1884613 to T2D [15, 16]. It is noteworthy that the minor allele frequencies among studies are significantly different. The G allele for rs1884613 had a frequency of 0.16 in diabetic Scandinavia [15], 0.18 in diabetic Norwegian [17], 0.27 in diabetic Ashkenazi Jewish [16], 0.48 in diabetic Japanese [10], and 0.43 in diabetic Chinese of the present study. The genetic predisposition to T2D in different populations may be related to the differences in allele frequencies.

There may be some unknown susceptibility functional variants that are in linkage disequilibrium (LD) with rs1884613 in the populations with positive findings. The conflicting results in other studies may be due to the varying LD patterns in different tested populations. Polymorphisms rs4810424, rs1884613, rs1884614, and rs2144908 were in almost complete LD in the Finnish and Ashkenazi Jewish populations; however the four polymorphisms are not situated in any confirmed functional regions [15], suggesting that these four polymorphisms may be in strong LD with a yet unknown but functional polymorphism [22]. The discrepancy among studies may also be attributable to the false-positive or false-negative results.

*HNF-4α* T130I polymorphism is a rare nonsynonymous variant that was shown to regulate* HNF-4α* gene expression and thus was shown to be associated with T2D risk [23, 24]. Furthermore, the interaction between* PPARG Pro12Ala* and* HNF-4α* rs2144908 was shown to postchallenge insulin secretion [25]. Trip3 gene was not shown to be a susceptibility gene of early-onset T2D in Japanese population but might play an important role in glucose metabolism through regulating the transcription activity of* HNF-4α* [26]. The above observations imply that the contribution of* HNF-4α* variants to T2D risk is not alone.

In order to confirm the association between rs1884613 and T2D susceptibility, we performed a meta-analysis among 8569 cases and 8528 controls from 8 studies including ours. Our meta-analysis showed there was no significant association between rs1884613 and T2D risk. Compared with the previous two meta-analyses [11, 17], the present one was involved in more studies. The meta-analysis by Johansson et al. only integrated results from homogenous populations of Scandinavians, although they detected a positive association but with a relatively small effect size (4000 cases and 7571 controls, OR = 1.14, *P* = 0.0004) [17]. The meta-analysis by Winckler et al. was involved with 7883 people and failed to confirm the significant association between rs1884613 and T2D risk (OR = 0.59, 95% CI = 0.90–1.06) [11].

There are some limitations to be noted. Firstly, we only genotyped rs1884613 under the hypothesis that rs1884613 may be in LD with other P2 promoter variants. It may not stand for the whole P2 promoter variants in the Han Chinese population, although previous findings have shown the P2 promoter variants are in high LD with each other in Europeans [16]; therefore the SNPs across the promoter and coding regions of* HNF-4α* with T2D and prediabetes should be further evaluated in future studies with different populations. Secondly, some populations in our meta-analysis consisted of the T2D cases from families with history of T2D. This may have confounded the results of the current meta-analysis. Thirdly, due to a lack of the detailed genotype frequency in most studies, the current meta-analysis only adopted the additive model that may not be the best heritable mode for this polymorphism. Fourthly, only a few studies had prediabetes subjects; therefore we were not able to perform the meta-analysis between rs1884613 and prediabetes risk. Last but not least, large heterogeneity was observed in the population-based subgroup meta-analysis. Since the hospital-based studies usually tend to have higher heterogeneity, the high heterogeneity in population-based studies may be due to the fact that some of the patients came from the families with history of T2D.

In conclusion, our results suggest that rs1884613 of* HNF-4α* is associated with prediabetes in Han Chinese population, whereas this polymorphism may not play an important role in the risk of T2D according to our case-control study and the updated meta-analysis. Future study is warranted to explore the LD patterns in the P2 region among the T2D patients from different ethnic populations.

## Figures and Tables

**Table 1:** Characteristics of the subjects included in this study.

Characteristic	Controls	Prediabetes	Type 2 diabetes	P1	P2	P3
Number of subjects	575	471	490			
Female/male	289/286	241/230	248/242	0.81	0.91	0.95
Age	57.94 ± 10.81	61.39 ± 11.43	62.76 ± 11.14	0.001	0.055	0.001
BMI	23.52 ± 3.17	25.28 ± 3.82	24.95 ± 3.46	0.001	0.143	0.001
Female						
Age	58.50 ± 10.00	61.66 ± 10.43	63.99 ± 10.28	0.001	0.01	0.001
BMI	23.18 ± 3.09	25.18 ± 4.04	24.69 ± 3.58	0.001	0.12	0.001
Male						
Age	57.38 ± 11.56	61.10 ± 12.42	61.51 ± 11.85	0.001	0.70	0.001
BMI	23.87 ± 3.22	25.37 ± 3.59	25.20 ± 3.32	0.001	0.59	0.001

Groups were compared using one-way ANOVA.

P1: Prediabetes versus controls; P2: type 2 diabetes versus Prediabetes; P3: type 2 diabetes versus controls.

**Table 2:** Comparison of genotypic and allelic distribution of rs1884613 in the (*HNF*)-*4α* gene among subjects with type 2 diabetes, Prediabetes, and healthy controls.

HNF4A rs1884613	CC	CG	GG	C	G	Additive model		Dominant model		Recessive model		HWE
						OR (95% CI)	*P*	OR (95% CI)	*P*	OR (95% CI)	*P*	
Controls	171	288	94	630	476							0.14
Prediabetes	128	224	117	480	458	1.40 (1.16–1.68)	0.0001^a^	1.47 (1.10–1.96)	0.008^a^	1.68 (1.22–2.32)	0.001^a^	
Type 2 diabetes	148	260	79	556	418	1.09 (0.82–1.32)	0.33	1.21 (0.92–1.60)	0.16	1.00 (0.70–1.41)	0.99	
Female	CC	CG	GG	C	G							
Controls	92	145	43	329	231							0.25
Prediabetes	71	108	60	250	228	1.48 (1.14–1.92)	0.003^a^	1.55 (1.04–2.30)	0.02	1.91 (1.20–3.04)	0.006^a^	
Type 2 diabetes	79	136	30	294	196	1.08 (0.54–1.43)	0.54	1.31 (0.89–1.94)	0.16	0.81 (0.47–1.38)	0.44	
Male	CC	CG	GG	C	G							
Controls	79	143	51	301	245							0.33
Prediabetes	57	116	57	230	230	1.33 (1.02–1.73)	0.035	1.41 (0.93–2.15)	0.09	1.50 (0.96–2.34)	0.07	
Type 2 diabetes	69	124	49	262	222	1.11 (0.85–1.44)	0.42	1.14 (0.77–1.71)	0.49	1.15 (0.73–1.82)	0.54	

^a^Significant results.

All of the ORs (95% CIs) and *P* values were adjusted for age and BMI.

**Table 3:** Comparison of minor allele frequency among studies.

Rs1884613	Minor allele frequency		*P* value	OR (95% CI)	Ethnicity
	Case	Control			
American Diabetes Association [12]	0.21	0.16	0.01	1.34 (1.07–1.66)	Scandinavia

Dupont and Plummer [13]	0.27	0.21	0.017	1.38 (1.06–1.80)	Ashkenazi Jewish

Silander et al. [15]	0.171	0.172	0.98	0.99 (0.73–1.33)	Caucasian

Boj et al. [8]	0.19	0.18	0.89	1.09 (0.75–1.59)	Discordant sibs
	0.12	0.17	0.25	0.67 (0.41–1.12)	Canada
	0.20	0.19	0.73	1.11 (0.89–1.39)	Scandinavia
	0.19	0.15	0.12	1.25 (0.99–1.58)	Sweden
	0.15	0.17	0.07	0.85 (0.73–0.99)	GCI USA
	0.17	0.18	0.66	0.93 (0.79–1.09)	GCI Poland

Winter [7]	0.482	0.469	0.71	1.05 (0.79–1.39)	Japanese

Love-Gregory et al. [16]	0.474	0.477	0.89	1.01 (0.88–1.15)	Japanese

Higgins and Thompson [14]	0.198	0.181	0.02	1.17 (1.03–1.35)	Norwegian

The present study	0.43	0.43	0.33	1.09 (0.82–1.32)	Chinese

GCI: Genomics Collaborative, Inc.

**Table 4:** The characteristics of each study included in meta-analysis.

Study	Country/racial decent	Publication year	Source of controls	Minor allele		Major allele		Sample size		(*HNF*)-*4α* rs1884613					
										Case			Control		
				Case	Control	Case	Control	Case	Control	CC	CG	GG	CC	CG	GG
American Diabetes Association [12]	Finland Scandinavia	2010	Family based and population based	0.21	0.16	0.79	0.84	787	410	498	254	35	291	105	14

Dupont and Plummer [13]	America Ashkenazi Jewish	1990	Family based	0.27	0.21	0.73	0.79	275	342	—	—	—	—	—	—

Silander et al. [15]	America Caucasian	2004	Hospital based	0.171	0.172	0.829	0.828	300	310	—	—	—	—	—	—

Boj et al. [8]	Discordant sibs	2001	Population based	0.19	0.18	0.81	0.82	609	580	—	—	—	—	—	—
	Canada			0.12	0.17	0.88	0.83	471	471	—	—	—	—	—	—
	Scandinavia			0.20	0.19	0.80	0.81	127	127	—	—	—	—	—	—
	Sweden			0.19	0.15	0.81	0.85	514	514	—	—	—	—	—	—
	GCI USA			0.15	0.17	0.85	0.83	1226	1226	—	—	—	—	—	—
	GCI Poland			0.17	0.18	0.83	0.82	1009	1009	—	—	—	—	—	—

Winter [7]	Japan Japanese	2003	Hospital based	0.482	0.469	0.518	0.531	192	192	59	81	52	53	98	41

Love-Gregory et al. [16]	Japan Japanese	2004	Hospital based	0.477	0.474	0.523	0.526	925	893	—	—	—	—	—	—

Higgins and Thompson [14]	Norway Scandinavia	2002	Population based	0.198	0.181	0.802	0.819	1644	1879	1048	507	67	1224	531	63

The present study	China Chinese	2014	Population based	0.43	0.43	0.57	0.57	490	575	148	260	79	171	288	94

Genomics Collaborative, Inc. (GCI).

**Table 5:** Meta-analysis of association between rs1884613 and type 2 diabetes risk under an additive model.

	Number of studies	Test of association			Test of heterogeneity		Test of publication bias	
	(Cases/controls)	OR (95% CI)	*Z*	*P*	*P*_heterogeneity_	*I* ^2^ (%)	Begg's	Egger's
All	8 (8569/8528)	1.05 (0.99–1.11)	1.92	0.06	0.03	54.7%	0.71	0.11
Asian	3 (1607/1660)	1.03 (0.92–1.14)	0.57	0.57	0.85	0%	0.60	0.36
White	5 (6962/6868)	1.06 (0.99–1.12)	1.81	0.07	0.005	73.3%	0.80	0.17
Hospital based	3 (1417/1395)	1.01 (0.90–1.13)	0.23	0.81	0.95	0%	0.60	0.84
Population based	5 (7152/7133)	1.15 (0.99–1.33)	1.94	0.05	0.005	72.9%	0.46	0.06
